# Quantum sensing of free radicals in macrophages reveals early autophagy-lysosome regulation in an atherosclerosis cell model

**DOI:** 10.1016/j.redox.2025.103825

**Published:** 2025-08-14

**Authors:** Siyu Fan, Han Gao, Wesley Nieuwhof, Thomas Mulder, Beatriz F.M. Fumelli, Runrun Li, Rokshana Sharmin, Maria Niora, Glaucia M. Machado-Santelli, Kirstine Berg Sorensen, Willy de Haan, Hélder A. Santos, Romana Schirhagl

**Affiliations:** a Department of Biomaterials and Biomedical Technology, University Medical Centre Groningen (UMCG), Antonius Deusinglaan 1, AV, 9713, Groningen, the Netherlands; b Department of Health Technology, Danmarks Tekniske Universitet, Ørsteds Plads, Denmark; c Department of Cell and Developmental Biology, Institute of Biomedical Science, Universidade de Sao Paulo, SP, Brazil

**Keywords:** Quantum sensing, Free radical, Nanodiamonds, NV centers

## Abstract

Atherosclerosis is the predominant cause of death in industrialized countries and is on track to become the foremost cause of death worldwide. Free radicals play a crucial role in regulating the degradation system in macrophages within plaques which lead to complications in atherosclerosis. They are linked to the decline of autophagy-lysosomes and plaque progression. However, their specific production sites and timing remain unclear. To investigate free radical production in the macrophage autophagy-lysosome system's response to stress, we employed nanodiamond-based quantum sensing. By incubating RAW 264.7 macrophages with nanodiamonds and oxidized low-density lipoprotein, we observed colocalization of nanodiamonds with oxLDL in the autophagy-lysosomal system. Quantum sensing was then applied to sense free radical production in the surrounding area. Our findings revealed a decrease in spin lattice relaxation (T1) times (more free radicals), with a 36.7 % and 31.8 % rise at 0.5 and 4 h of oxLDL incubation compared to controls. Additionally, we observed the nuclear translocation of transcriptional factor EB (TFEB), the master transcriptional regulator of autophagy-lysosomal biogenesis. This suggests the initiation of an autophagy-lysosomal program, which enhanced the cell's degradative capacity. Consequently, T1 values firstly increased after 1h. At 4h, a significant TFEB nuclear translocation was observed, leading to the increase of T1 values by 8.2 % and 20.3 % at 6 and 8 h, respectively, compared to the 4-h mark.

## 1 Introduction

Atherosclerosis is the primary cause of death in industrialized nations and is expected to become the leading cause globally in the near future [1]. Macrophages play a key role in the progression of atherosclerosis. They ingest oxidized low-density lipoprotein (oxLDL) and cellular debris, form foam cells, and contribute to inflammation and dysfunction within the plaque [2,3]. Investigating the cellular processes behind macrophage dysfunction is crucial for scientific understanding and clinical advancements of atherosclerosis. However, many questions remain about the mechanisms by how oxLDL triggers macrophage dysfunction. One of the key factors in this process is the interaction between free radical generation and autophagy-lysosome biogenesis.

It has been shown that macrophage autophagy plays a protective role in atherosclerosis [2]. Autophagy is a highly conserved process that is essential for degrading and recycling long-lived or damaged intracellular material, including accumulated lipids [2,4,5]. Disruption of macrophage autophagy in mice increase atherosclerosis significantly [6]. The observed effects include reducing lipophagy, hyperactivating inflammasome and impairing lysosome-mediated cholesterol efflux [6].

Impaired autophagy also leads to mitochondrial dysfunction, triggering apoptosis through free radicals [7,8]. This apoptosis of foam cells is crucial in forming the lipid-rich necrotic core in plaques and contributes to plaque rupture [9]. These plaques lead to clogging of blood vessels and thus severe complications for patients, including myocardial infarction, stroke or even death [10].

Several mechanisms have been proposed for the involvement of free radicals in atherosclerosis. Evidence for the involvement of free radicals (especially in the early stage of the disease) has been reported via measuring lipid peroxidation, a consequence of free radical damage [11]. Such lipid peroxidation occurs when free radicals degrade low density lipoproteins. These oxidation products are ingested by cells which leads to foam cell and plaque formation [12]. However, attempts to prevent free radical generation lead to mixed results [13,14]. The interaction between free radicals and dysfunction in macrophage autophagy-lysosome processes remains unclear.

The transcription factor EB (TFEB), part of the microphthalmia subfamily, serves as the master regulator of autophagy-lysosomal biogenesis genes, enhancing the cells' overall degradative capacity and lysosomal lipid catabolism [15,16]. Its expression and nuclear translocation are linked to cellular oxidative stress levels [16]. Although previous studies have detected changes in related proteins, these methods typically do not clarify the relationship between free radicals and autophagy-lysosomal biogenesis in atherosclerotic macrophages, and they do not allow for real-time measurement. Additionally, organic dye-based methods for detecting cellular reactive oxygen species (ROS) are problematic due to the dye bleaching over time, which complicates tracking ROS changes [17,18]. These methods are also irreversible, measuring accumulated ROS production rather than the current free radical concentration.

Quantum sensing, based on NV centers in fluorescent nanodiamonds (FND), offers an attractive tool to measure free radicals at the nanoscale [19,20]. These NV centers emit exceptionally stable red fluorescence, which changes in intensity based on the electron spin state [[21], [22], [23], [24]]. Consequently, this fluorescence can be used to measure magnetic fields or magnetic noise [[25], [26], [27]]. This technique has recently been applied to detect free radical generation in living cells, including in studies of stress responses in immune cells [28,29], cancer cells [30] and yeast cells [31]. It has been also used in bacteria [32], or virus-affected cells [33].

**Fig. 1:**
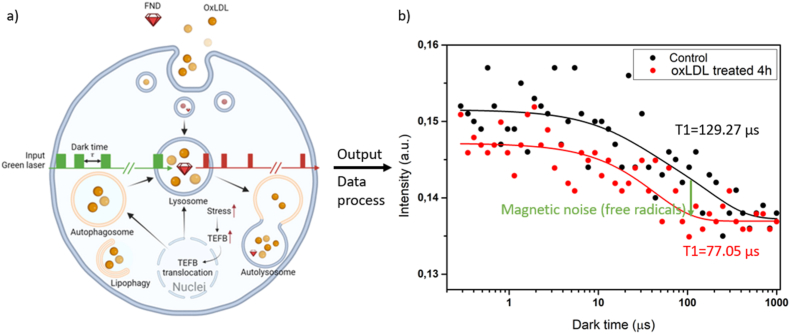
a) Applying T1 relaxometry in oxLDL-treated macrophages. Fluorescent nanodiamonds (FND) and oxLDL were fed to macrophages simultaneously. After a certain incubation period, T1 measurements were conducted on live cells to assess free radical production at specific sites. Created with BioRender.com b) The emitted red fluorescence signal pulses from FND were collected and fitted to a biexponential curve. The T1 value, extracted from this curve, represents the amount of magnetic noise (in this case caused by free radicals). A lower T1 value indicates a higher concentration of free radicals.

In this study, we demonstrate nanodiamond-based free radical quantum sensing in an atherosclerosis cell model. We measured changes in free radical levels in oxLDL-treated macrophages, with varying levels corresponding to different oxLDL treatment times. Additionally, we examined the expression and nuclear translocation of TFEB to link lysosomal free radical levels with autophagy-lysosome biogenesis. A schematic outline of the experiments in this article are shown in Fig. 1.

## 2 Results and discussion

### 2.1 Evaluating oxLDL levels, cell viability and oxidative stress levels in macrophage-derived foam cell formation

**Fig. 2:**
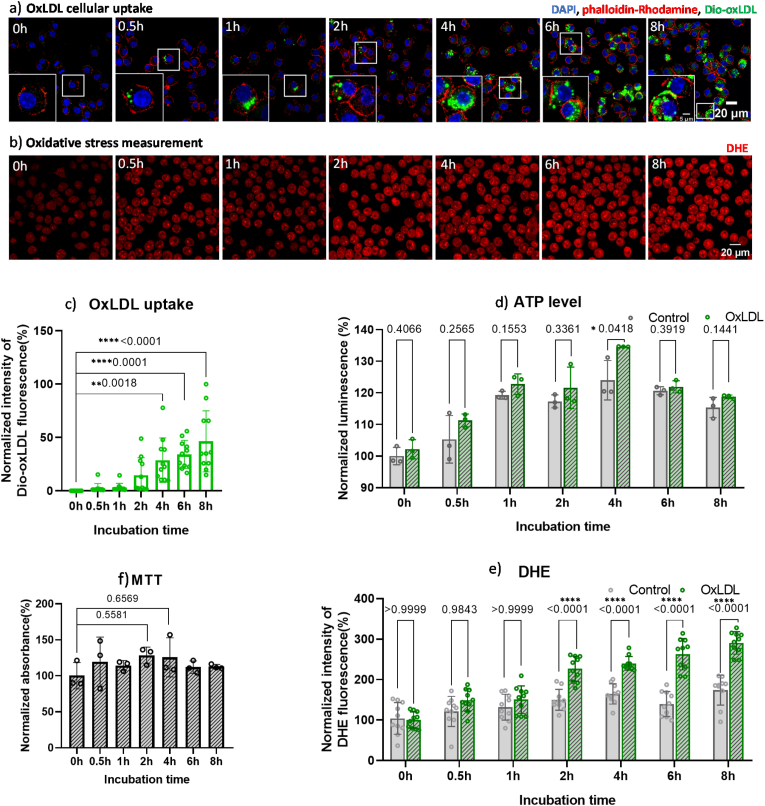
OxLDL accumulation and cellular ATP levels in RAW 264.7 macrophages. Macrophages were incubated with 50 μg/mL oxLDL for 0, 0.5, 1, 2, 4, 6 and 8h. **a)** Imaging: OxLDL was conjugated with Dio (green), nuclei were stained with DAPI, and the cell skeleton was stained with phalloidin-Rhodamine. **b)** Representative images of intracellular DHE signal distribution, revealed the ROS levels. **c)** Quantification of OxLDL: The average fluorescent intensity of Dio-oxLDL per cell was quantified using FIJI, with a total of 60 cells per group analyzed. **d)** ATP Measurement: Cellular ATP levels were determined using the Cell Titer assay. **e)** Quantification of DHE fluorescence intensity. The average fluorescent intensity of ROS per cell was quantified using FIJI, with a total of 60 cells per group analyzed. **f)** Cellular viability was measured by MTT assay. Data are expressed as means ± SD from 3 independent experiments, normalized by the mean value of 0h control group. Statistical significance was evaluated using one-way ANOVA for c) and *t*-test for d), e) and f).

To evaluate the cellular uptake of oxLDL, RAW 264.7 macrophages were incubated with Dio-conjugated oxLDL (green fluorescent, Fig. 2a). Dio-oxLDL uptake increased significantly over time, with notable rises observed from 4 h onwards (∗∗*p* = 0.0018) (Fig. 2c). This uptake is mediated by scavenger receptors (SRs) like SR-A and CD36 [[34], [35], [36]], which facilitate excessive accumulation of oxLDL, leading to foam cell formation due to the lack of negative regulatory receptor feedback [34,37,38] (∗∗∗∗*p* = 0.0001 at 6 h and ∗∗∗∗*p* < 0.0001 at 8 h).

The cell viability was assessed by measuring the cellular ATP levels after treating macrophages with 50 μg/mL oxLDL for varying durations. As reported previously, this concentration effectively promotes lipid accumulation without causing significant cytotoxity [39]. This is also confirmed by MTT assay in Fig. 2f, no significant change was found as OxLDL incubation time increased. Compared to the control, the ATP levels in the oxLDL-exposed group were slightly higher. This tendency remained until 4 h, showing a significant increase there (∗p = 0.0418), then decreased at 6h and 8h. (Fig. 2d).

To understand the change of cell viability, we also assessed the reactive oxygen species levels as the presence of ROS is part of the proposed mechanisms for foam cell apoptosis [40]. To measure ROS levels in macrophages, we used dihydroethidium (DHE), which is easily oxidized by oxidants like superoxide to produce 2-hydroxyethidium. This compound then binds with DNA and emits red fluorescence [41].

As shown in Fig. 2b, RAW 264.7 macrophages treated with oxLDL for various durations displayed strong nuclear red fluorescence starting from 2 h. Quantification in Fig. 2e revealed steady ROS levels before 2 h. From 2 h, a rise (∗∗∗∗*p* < 0.0001) of ROS was observed. The high cellular ROS level remained until 8h. The observed fluctuation in ROS levels suggests a correlation between cell viability and ROS production. At 2 h, since mitochondria are the energy hubs of the cell, they are also major producers of ROS due to electron reactions with oxygen [42], which leads to the alignment of ATP and ROS levels. After that, the continuous stress exposed to the cells resulted in a decrease of viability.

### 2.2 Cellular uptake and localization of particles in macrophages

**Fig. 3:**
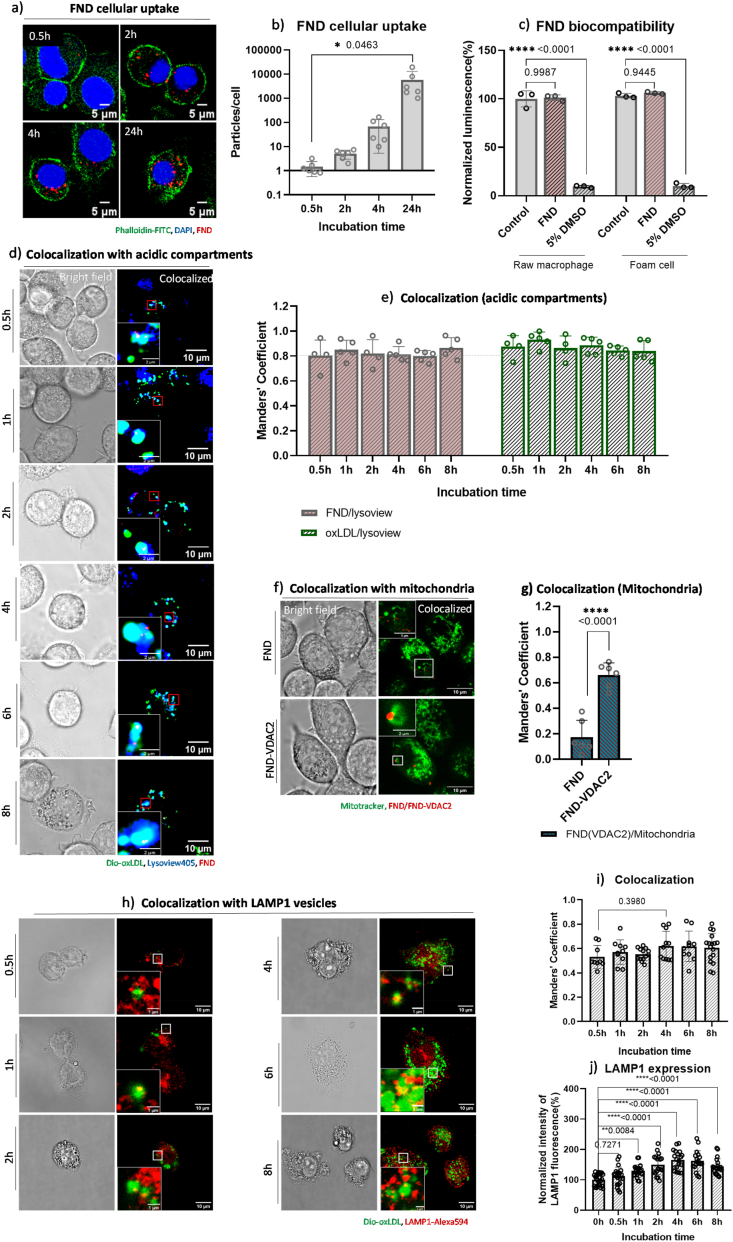
a) Representative images of FND uptake in RAW 264.7 macrophages. Cells were incubated with 10 μg/mL FNDs for 0.5, 2, 4 or 8 h. Nuclei: DAPI (blue); Actin filaments: Phalloidin-FITC (green); FND (red). b) FND particles counting, in total 60 cells per group were analyzed by FIJI. c) biocompatibility of FND in macrophages and foam cells was assessed through an ATP assay. d) Intracellular location of FND and oxLDL in RAW 264.7 macrophages. Macrophages were incubated with FNDs (red) and Dio-oxLDL (green) for 0.5, 1, 2, 4, 6, and 8 h, then stained by lysoview 405 (blue) and imaged. e) Quantitative colocalization analysis of FND with acidic organelles and oxLDL with acidic organelles at various incubation times. A total of 60 cells per group were analyzed. f) RAW 264.7 macrophages were incubated with FND-VDAC2 (red) for 2h. Then cells were stained with Mitotracker Green and imaged by Leica SP8x confocal microscope. g) Quantitative colocalization analysis of FND-VDAC2 with mitochondria after 2h incubation. A total of 60 cells per group were analyzed. h) Intracellular colocalization of oxLDL LAMP1, LAMP1 was labelled by Alexa 594 (red) (for separate channels see Supplementary Fig. S4). I) Mander's coefficient analysis from panel h). j) Quantification of LAMP1 expression by measuring mean fluorescence signal. A total of 60 cells per group were analyzed. Data are expressed as means ± SD from 3 independent experiments, c) was normalized by the mean value from the control group. Statistical significance was evaluated using a one-way ANOVA for b), c), e), i) and j). T-test was used for g).

As shown in Fig. 3a, the uptake of FND particles by RAW 264.7 macrophages exhibited a time-dependent increase over 8 h, this observation was confirmed by quantification in Fig. 3b, consistent with previous reports [29]. To assess the effect of FND on macrophages or oxLDL-derived foam cell, cell viability was measured using Cell Titer, with 5 % DMSO as a positive control (Fig. 3c). After 24 h of incubation, 5 % DMSO showed significant cytotoxicity (∗∗∗∗*p* < 0.0001), while no significant change was observed in FND-treated macrophages or foam cells. This indicates that FNDs exhibit good biocompatibility, ensuring that their presence does not interfere with oxidative stress measurements in subsequent experiments.

To investigate the localization of FND and oxLDL after endocytosis by macrophages, confocal images of live cells were taken following co-incubation with FNDs and Dio-oxLDL for 0.5, 1, 2, 4, 6, and 8 h. Lysoview 405 was used to track acidic organelles such as lysosomes. As shown in Fig. 3d, colocalization of FND and Dio-oxLDL within acidic organelles was already evident at 0.5 h. By 4 h, the organelle swelling was observed, likely due to the overaccumulation of oxLDL. Previous studies indicate that only about 50 % of oxLDL can be effectively degraded by lysosomes, partly due to their resistance to lysosomal proteolytic activity [40,43]. This defective hydrolysis results in oxLDL being trapped within the lysosomal compartment.

The colocalization of FND with acidic compartments and oxLDL with acidic compartments was analyzed using FIJI, with z-stack confocal images. The overlapping fraction of different components was quantified using Mander's coefficients [[44], [45], [46]]. Fig. 3e shows that approximately 80 % of FND colocalized with acidic compartments, with no statistically significant change over 8 h. Similarly, oxLDL was highly colocalized with acidic compartments, with a slight decrease in the colocalization fraction from 1 h (0.931 ± 0.061) to 8 h (0.839 ± 0.084) (Table S1), though this was not statistically significant (*p* = 0.3870). These results confirmed that FND-based free radical measurements were performed in lysosomes containing oxLDL at the corresponding incubation times.

To further confirm the colocalization of Dio-oxLDL with lysosomes, we stained LAMP1 [47](Fig. 3h). Colocalization analysis confirmed the location of Dio-OxLDL (Fig. 3i), over 50 % of OxLDL were overlapped with LAMP1 signal, and as the incubation increased to 4h, this number increased to around 63 %. The expression level of LAMP1 was quantified in Fig. 3j), a significant increase of LAMP1 level was found since 1h incubation, this suggested the uptake of oxLDL upregulated lysosome abundance to enhance the degrative capacity, which supported our original hypothesis.

Additionally, following previous protocol [29,48,49], we coated the FND with voltage dependent anion channel 2 antibody (*anti*-VDAC2), This coating targets FNDs to the outer membrane of mitochondria. As shown in Fig. 3f, after 4hrs incubation, some particles have reached the mitochondria. Mander's coefficient analysis (Fig. 3g) revealed that ∼65 % of FND-VDAC2 particles colocalized with mitochondria, a significant increase compared to uncoated FND (∗∗∗∗p < 0.0001).

### 2.3 Free radical measurement at different sites

**Fig. 4:**
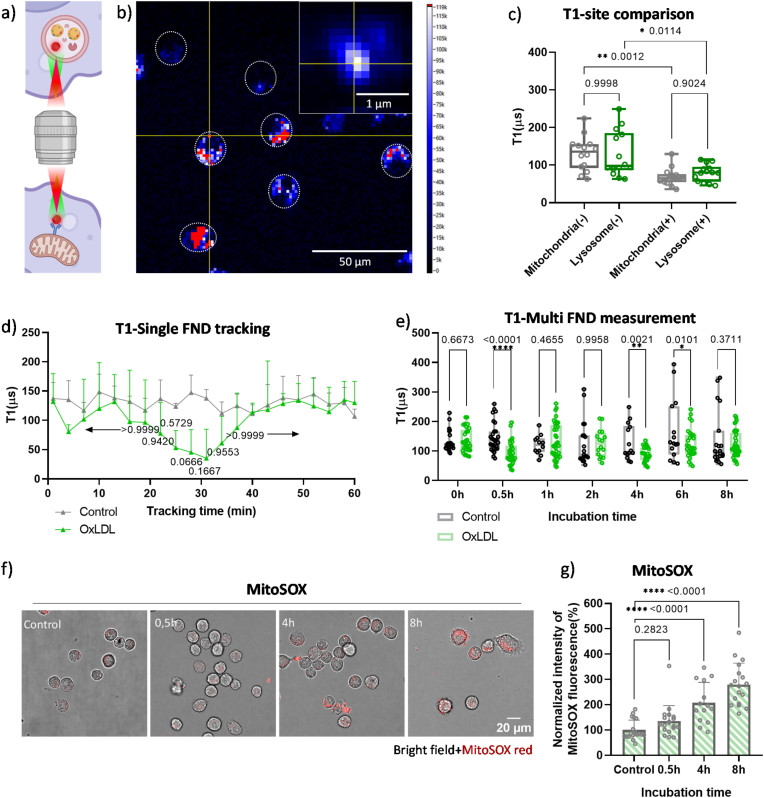
a) Schematic illustration of performing free radical detection by T1 relaxometry in different organelles, Created in https://BioRender.com. b) A representative fluorescence image of FNDs within macrophages. The white dashed circle outlines the cell border. An intensity bar (photon counts/s) is shown on the right. The inset in the top right corner is a zoomed-in view of the FND particle located at the yellow cross (6 × 10^6^ counts/s). c) T1 was performed near mitochondria after incubating ox-LDL with cells for 4h (10 μg/mL FND-VDAC2 was added in between to have 2-h incubation time). A total of 15 FND-VDAC2 particles were measured. The effect of VDAC2 coating on T1 has been ruled out in a previous paper [48]. (−) = no oxLDL, (+) = cells were treated with oxLDL. d) Real-time T1 tracking of a single FND within a macrophage. T1 values were recorded continuously every 2 min for 60 min. Data are expressed as means ± SD from 3 independent experiments. Statistical significance was determined using a two-way ANOVA. e) T1 measurements from multiple FNDs in macrophages after incubation with oxLDL for specific time intervals. A total of 15 FNDs were measured. Whiskers indicate the range of data points from minimum to maximum. The line within the box represents the median value, while the upper and lower edges of the box denote the higher and lower quartiles, respectively. Data are presented from 3 independent experiments. Statistical significance was determined using a two-way ANOVA. (f) shows conventional ROS measurements with MitoSOX Red overlayed with brightfield images (for separate channels see Supplementary Fig. S5) which are further quantified in (g).

Using NV centers, FNDs sense surrounding free radical production in real-time without causing additional cytotoxic effects. As illustrated in Fig. 4a, we measured the real-time free radical production in different cellular sites, autolysosome and mitochondria. Fig. 4b shows a typical T1 measurement window, where endocytosed FNDs (red) were visible in macrophages (dashed circle). Once a single FND was located in a cell (top right corner in Fig. 4b), T1 measurements were conducted. Each measurement involved 10,000 repetitions of laser pulsing, taking approximately 2 min to complete. The emitted red fluorescence pulses were plotted and analyzed using a biexponential decay (Fig. 1b). As opposed to single exponential models which are used for single NV center measurements, this bi-exponential model was found empirically earlier to better predict concentrations when using nanodiamonds with large ensembles of NV centers [50]. A potential explanation might be the different environment NV centers are in within a nanodiamond caused by the proximity of different surfaces, dangling bonds or internal defects [50,51]. The T1 value, derived from the decay rate of the biexponential curves, reflected surrounding magnetic noise [51]. Smaller T1 values indicated higher radical concentrations. While charge transfer from NV^−^ to NV^0^ has proven to cause potential artifacts in single NV center measurements and measurements with high laser powers, we never observed this phenomenon under our measurement conditions [52,53].

Fig. 4d shows the T1 tracking of a single FND over 60 min. Compared to the control, the oxLDL-treated macrophage exhibited a decrease in T1 at 27 min (*p* = 0.0666), indicating free radical production likely due to oxLDL endocytosis, as 87.4 % of oxLDL colocalized with lysosomes (Fig. 3e). Free radical levels then stabilized to control levels after 40 min. To confirm this observation, additional 1-min measurements were performed on different FNDs at specific oxLDL incubation time points. Fig. 4e showed T1 values from 15 FNDs, revealing a significant increase in free radicals at 0.5h (∗∗∗∗*p* < 0.0001), 4h (∗∗*p* = 0.0021), and 6h (∗*p* = 0.0101). At 4 h, the mean T1 value for the experimental group is 88 μs, compared to 129 μs for the control group, indicating a 31.8 % reduction. Similarly, at 6 h, there's a 23.6 % reduction of T1 value and for 8 h 11.5 % reduction.

These data indicate that while oxidative stress is present (as evidenced by lower T1 values in the experimental group compared to controls), the degree of stress decreases over time. Specifically, if comparing with 4h, from 4h to 6h, the stress level decreased 8.2 %, for 8h, this number decreased 20.3 %. As oxLDL was accumulated in lysosomes, lipid peroxidation (LPO), a free-radical chain reaction involving unsaturated fatty acids, could occur. This would result in unstable lipid peroxides, amplifying oxidative stress and potentially accelerating atherosclerosis [[54], [55], [56]]. The increase of lysosomal LPO levels in early foam cells’ transformation (6h) was also observed by Zhang, et,al. [54], aligning with our observations.

To compare the oxidative stress in different organelles, mitochondrial-specific targeting was achieved using FNDs coated with *anti*-VDAC2 (FND-VDAC2), a mitochondrial protein [29,48,49]. Previous studies have confirmed that this coating by itself does not affect T1 measurements [49]. After delivering FND-VDAC2 to mitochondria, T1 measurement was then performed after incubating cells with oxLDL for 4h. A significant decrease of T1 value was observed in mitochondria, indicating that mitochondria were stressed by cellular oxLDL accumulation. However, no statistically significant difference was found between the T1 values of mitochondria and lysosomes (p = 0.9024, Fig. 4c). To further quantify the oxidative stress, free radical concentrations were estimated using a calibration curve [57], At 4 h, the calculated free radical concentration was ∼228.85 nM for mitochondria and ∼116.15 nM.for lysosomes. This suggests that at 4h, on average, mitochondria generate more free radicals than lysosomes under oxLDL-induced stress. Here it is also worth noting that we are underestimating the real effect here due to the fact that not all particles are successfully attached to mitochondria.

To validate these findings, we used MitoSOX Red, a mitochondrial ROS-specific assay. As shown in Fig. 4f and g, MitoSOX Red confirmed the increase in mitochondrial ROS at 4 h, consistent with the T1 measurements. Moreover, mitochondrial ROS levels continued to rise until 8 h, which correlated with the overall cellular ROS levels measured by DHE staining. Interestingly, neither MitoSOX Red nor DHE could detect a significant increase in lysosomal free radicals at early time points (0.5 h). To further investigate it, we have performed additional experiments using the FND-VDAC2 sensor at 0.5 h and 1 h post-treatment to assess mitochondrial ROS levels. These data are now presented in Fig. S8. Our results show that mitochondrial ROS levels begin to rise as early as 0.5 h, with a further increase at 1 h and a peak at 4 h. This time-course supports the idea that mitochondria also contribute significantly to early redox signaling events. This highlights the higher site sensitivity of T1 relaxometry, which allows for the detection of oxidative stress in lysosomes even at early stages.

### 2.4 TFEB and autophagy marker RAB11a expression

The accumulation of oxLDL in cells and the fluctuation of free radical levels in lysosomes prompted us to investigate autophagic degradation, particularly the expression of TFEB. TFEB is the predominant transcription factor responsible for the coordinated expression of autophagy-lysosomal genes and the pro-degradative response in some cells like macrophages [58,59]. Under normal conditions, TFEB is phosphorylated by the *mammalian target of rapamycin* (mTOR) complex 1 (mTORC1) kinase and stayed in the cytoplasm [15,16].

**Fig. 5:**
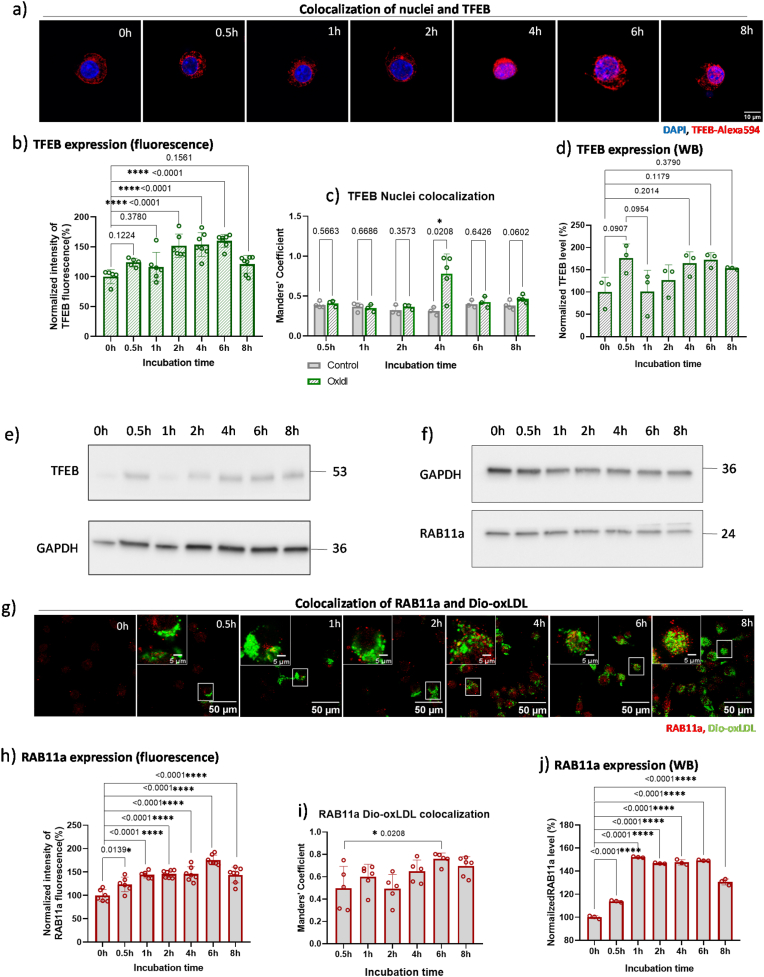
a) TFEB was labelled after RAW 264.7 macrophages were incubated with oxLDL for different time. Red: TFEB (Alexa 594); Blue: Nuclei (DAPI) (for separate channels see Supplementary Fig. S6). b) Quantification of TFEB expression per cell by calculating fluorescence intensity. Data are expressed as means ± SD from 3 independent experiments and normalized by defining the mean value of 0h control as 100 %. Statistical significance was determined using one-way ANOVA. c) Colocalization analysis of TFEB and nuclei. A total of 60 cells per group were analyzed. Data are presented from 3 independent experiments. Statistical significance was determined using a *t*-test. d) PAGE-Western Blotting (WB) quantification of TFEB expression after incubating cells with oxLDL for different time. Data are expressed as means ± SD from 3 independent experiments, normalized by defining the mean value of 0h control as 100 %. Statistical significance was evaluated using a one-way ANOVA. e) PAGE-Western Blotting (WB) of TFEB expression. f) PAGE-Western Blotting of RAB11a expression after incubating cells with oxLDL for different time. g) RAW 264.7 macrophages were incubated with Dio-oxLDL (green) for different time point (from 0.5h to 8h). Then cells were fixed and stained with RAB11a antibody (labelled with Alexa 594, red) and imaged by Leica SP8x confocal microscope. h) The average RAB11a (Alexa 594) fluorescent intensities per cell were analyzed by FIJI. Data are expressed as means ± SD from 3 independent experiments and normalized by defining the mean value of 0h control as 100 %. A total of 60 cells per group were analyzed. i) Quantitative colocalization analysis of the percentage of Dio-oxLDL that was overlapped with RAB11a-positive sites. A total of 60 cells per group were analyzed. Data are presented from 3 independent experiments. Statistical significance was determined using a one-way ANOVA. j) Quantification of RAB11a expression. Data are expressed as means ± SD from 3 independent experiments, normalized by defining the mean value of 0h control as 100 %. Statistical significance was evaluated using a one-way ANOVA.

In RAW 264.7 macrophages incubated with oxLDL, TFEB translocation to the nucleus was observed at 4 h (Fig. 5a). Colocalization analysis confirmed this observation, showing a significant increase (∗*p* = 0.0208) (Fig. 5c). For macrophages incubated with oxLDL, TFEB expression showed a slight increase at 0.5 h without significant difference. From 2h, the increase became significant until 6 h (∗∗∗∗*p* < 0.0001), after which it decreased again (Fig. 5b). To confirm the imaging results, Western blot results of TFEB nuclear protein levels are shown in Fig. S3 in the supplementary information. Western blot analysis (Fig. 5d–e) corroborated these fluorescence-based observations. From the mechanistic side, the overaccumulation of oxLDL in lysosomes (Fig. 2a) could potentially induce lysosomal oxidative stress (Fig. 4e), causing TFEB to be dephosphorylated and translocated to the nucleus, where it induced the transcription of target genes. This dephosphorylation could be mediated by proteins such as stress-activated phosphatase 2A (PP2A) as reported before [60]. In addition to that, oxidative stress induces a redox switch in TFEB itself by oxidizing Cys 212 within its 14-3-3–binding motif and promotes intermolecular disulfide bond formation and TFEB oligomerization [61].

The activation of TFEB might lead to autophagy-lysosomal biogenesis, as the fused shape of lysosomes was observed in Fig. 3d. This likely resulted from the fusion of autophagosomes and lysosomes to form autolysosomes. LAMP1 colocalization in Fig. 3h also supported the hypothesis. To further investigate autophagosome activation, we checked the expression level of RAB11a, as RAB11a-positive membranes are primary direct platforms for autophagosome formation [62]. As shown in Fig. 5g–h, RAB11a expression significantly increased from 1 h and continued to rise until 8 h. This is also confirmed by Western Blot analysis in Fig. 5f–j. Colocalization analysis of RAB11a with oxLDL (labelled by Dio) revealed that over 50 % of oxLDL localized to RAB11a-positive sites at 0.5 h (Fig. 5i). Mander's coefficients remained above 50 % at subsequent time points, with a significant increase between 2 and 4 h, possibly linked to TFEB nuclear translocation. This change explained the gradual reduction in free radical levels in lysosomes after 0.5 h (Fig. 5d), suggesting that autophagy-lysosomal biogenesis helps rescue lysosomal dysfunction. However, due to ongoing oxLDL endocytosis, the accumulated oxLDL eventually leads to the increase of ROS level (Fig. 2, Fig. 3, Fig. 4g) and decrease of cell viability (Fig. 2d), resulting in the transformation of macrophages to foam cells.

## 3 Conclusion

Despite the well-established role of free radicals in atherosclerosis, measuring them selectively at the subcellular level in real-time has been challenging due to technical limitations. In this study, we used a radical-specific detection method to demonstrate that oxLDL accumulation significantly decreases T1 values (indicating more free radicals) in lysosomes at 0.5 and 4 h, compared to controls.

By performing continuous real-time T1 measurements, we captured localized oxidative stress dynamics specifically within autolysosomes, where oxLDL is accumulated. This provides direct insight into the role of free radicals in lysosomal dysfunction during oxLDL accumulation.

We further compared T1 measurements across different organelles and found different oxidative stress responses in mitochondria and autolysosomes. Interestingly, lysosomal oxidative stress appears to trigger a protective response via TFEB activation, the master regulator of lysosome biogenesis and cellular homeostasis. At 4 h, TFEB showed significant nuclear translocation, suggesting an adaptive response to lysosomal dysfunction. Following this, T1 values in autolysosomes increased by 8.2 % at 6 h and 20.3 % at 8 h, compared to 4 h, indicating a reduction in local oxidative stress. However, due to ongoing oxLDL endocytosis, the overall cellular stress—particularly in mitochondria—remained high.

In conclusion, our study extends the application of nanodiamond‐based T1 relaxometry to an early atherosclerosis model. We demonstrate a time-resolved quantification of lysosomal oxidative stress in oxLDL‐loaded macrophages, and link this to TFEB-mediated autophagy–lysosomal biogenesis. Earlier studies measured ROS in large cell populations or based on indirect measurement [17,63,64], here, we combine FNDs with autolysosomal markers (Lysoview 405, LAMP1, RAB11a) and perform T1 measurements to discriminate autolysosomal vs. mitochondrial stress. This is a specificity that has not been previously demonstrated. From biological insight, we showed an early burst of lysosomal ROS triggers TFEB nuclear translocation, compared to earlier work, our approach offers higher throughput, better site specificity, and a direct link between quantum‐level stress measurements and key cellular stress-response pathways. Future studies will be necessary to investigate the upstream signaling mechanisms that regulate TFEB activation in response to oxLDL, including the potential involvement of PP2A and TFEB phosphorylation. These directions represent important avenues for further dissecting the molecular link between lipid uptake, redox imbalance, and lysosomal biogenesis in macrophages.

These findings lay the foundation for future investigations of free radicals at the nanoscale and for assessing therapeutic strategies aimed at reducing cellular or bloodstream free radical burden.

## CRediT authorship contribution statement

**Siyu Fan:** Writing – review & editing, Writing – original draft, Data curation, Conceptualization. **Han Gao:** Writing – review & editing, Data curation. **Wesley Nieuwhof:** Writing – review & editing, Data curation. **Thomas Mulder:** Writing – review & editing, Data curation. **Beatriz F.M. Fumelli:** Writing – review & editing, Data curation. **Runrun Li:** Writing – review & editing, Data curation. **Rokshana Sharmin:** Writing – review & editing, Data curation. **Maria Niora:** Writing – review & editing, Data curation. **Glaucia M. Machado-Santelli:** Funding acquisition, Project administration, Supervision, Writing – review & editing.**Kirstine Berg Sorensen:** Writing – review & editing, Supervision, Resources. **Willy de Haan:**Writing – review & editing, Data curation. **Hélder A. Santos:** Writing – review & editing, Supervision. **Romana Schirhagl:** Writing – review & editing, Supervision, Funding acquisition.

## Declaration of competing interest

R.S. is founder and shareholder of QTsense, a company which commercialises quantum sensing equipment.

## Data Availability

Data will be made available on request.
