# Supplementary material for: Quantum sensing of free radicals in macrophages reveals early autophagy-lysosome regulation in an atherosclerosis cell model
Source: Redox Biol. 2025 Aug 14;87:103825. doi: 10.1016/j.redox.2025.103825 (PMC12628021; doi:10.1016/j.redox.2025.103825)
Supplement: Multimedia component 1 [file mmc1.docx]

**Supplementary Information**

**Quantum Sensing of Free Radicals in Macrophages Reveals**

**Early Autophagy-lysosome regulation in Atherosclerosis**

Siyu Fan^1^, Han Gao^1,2^, Wesley Nieuwhof^1^, Thomas Mulder^1^, Beatriz Fumelli^1^, Runrun Li^1^, Rokshana Sharmin^1^, Maria Niora^3^, Kirstine Berg Sorensen^3^, Willy de Haan^1^, Hélder A. Santos^1,2^, Romana Schirhag^1^

1. Department of Biomaterials and Biomedical Technology, University Medical Centre Groningen (UMCG), Antonius Deusinglaan 1, 9713 AV, Groningen, The Netherlands.
2. Drug Research Program, Division of Pharmaceutical Chemistry and Technology, Faculty of Pharmacy, University of Helsinki, FI-00014 Helsinki, Finland.
3. Department of Health Technology, Danmarks Tekniske Universitet, Ørsteds Plads, Denmark.

**Material and methods**

**Materials**

FND particles (Adamas Nanotechnologies, North Carolina, USA) have a hydrodynamic diameter of 70 nm and contain over 300 nitrogen-vacancy (NV-) centers per particle (according to the manufacturer). These particles are produced through high-pressure high-temperature synthesis followed by high-temperature annealing^1^. They undergo a cleaning process in oxidizing acid to achieve an oxygen-terminated surface^2^. This specific size of FNDs was selected due to their brightness, ease of tracking, and high NV center count, which ensures a favorable signal-to-noise ratio. Larger particles were avoided as the NV centers would be too distant from the diamond surface, reducing measurement effectiveness. Each measurement in this study averaged the signals from all NV centers in a particle, enhancing signal reliability. The biocompatible FNDs maintain stable fluorescence after cellular uptake, making them suitable for biological applications^3,4^.

OxLDL (oxidized low-density lipoprotein) and Dio-oxLDL were purchased from Thermo Fisher (ThermoFisher, Netherlands).

**Diamond preparation**

Antibody conjuagtion was established in prior research ^5^. In this study, 0.1 mg/mL anti-VDAC2 (GeneTex, The Netherlands) were mixed with 10 μg/mL FNDs at a 1:4 ratio, vortexing for 1 minutes, then incubated at room temperature for 10 minutes, resulting in the formation of FND-anti-VDAC2.

**Cell culture**

RAW 264.7 macrophages were sourced from the American Type Culture Collection (ATCC, Manassas, VA) and cultured in DMEM with 10% FBS, 100 U/ml penicillin, and 100 U/ml streptomycin. The cells were maintained at 37°C in a 5% CO_2_ atmosphere (all reagents from Invitrogen, Carlsbad, CA).

**Particle uptake in macrophages**

To analyze oxLDL uptake, 50,000 cells/mL were seeded in 35 mm glass bottom Petri dishes (Grenier Bio-one, Germany)and incubated with 50 μg/mL Dio-oxLDL for various durations (0, 0.5, 1, 2, 4, 6, or 8 hours) at 37°C with 5% CO_2_. After incubation, the medium was removed, cells were washed with 1× PBS, fixed with 3.7% formaldehyde, and stained with DAPI and Rhodamine-phalloidin (Abcam, UK). Z-stack confocal images were taken with a Leica SP8x microscope (Leica, Germany), detecting FNDs at 561/659 nm, DAPI at 358/461 nm, and Rhodamine at 550/590 nm.

Z-stack images covering the entire cell volume were obtained, and approximately 60 randomly selected cells from each of three independent experiments were analyzed using FIJI to measure mean fluorescence intensity per cell. A control group was used to subtract background values from other experimental groups.

For FND uptake analysis, 70 nm FNDs were sonicated for 10 minutes. Cells (50,000 cells/mL) were seeded in 35 mm glass bottom Petri dishes and incubated with 10 μg/mL FNDs for 0.5, 2, 4, or 8 hours at 37°C with 5% CO_2_. After incubation, the FND-containing medium was removed, cells were washed with 1× PBS, fixed with 3.7% formaldehyde, and stained with DAPI and Fluorescein Isothiocyanate (FITC)-phalloidin (Abcam, UK). Z-stack confocal images were taken with a Leica SP8x microscope (Leica, Germany), detecting FNDs at 561/659 nm, DAPI at 358/461 nm, and FITC at 495/510 nm.

**Subcellular location of FNDs and oxLDL in macrophages**

To determine the intracellular location of diamond particles and oxLDL within macrophages at various incubation times, Lysoview 405 (Biotium, USA) was used to label lysosomes. Cells were seeded at a density of 50,000 cells/mL in 35 mm glass bottom Petri dishes and incubated with 10 µg/mL sonicated FNDs and 50 µg/mL Dio-oxLDL for different durations (0, 0.5, 1, 2, 4, 6, or 8 hours) at 37°C with 5% CO_2_. After incubation, cells were washed with PBS to prevent continuous FND uptake, enabling the tracking of previously endocytosed FNDs. Lysoview 405 (diluted 1000 times from stock) was then added, and cells were incubated for 10-30 minutes. Live cell imaging was performed using a SP8x Leica confocal microscope.

Approximately 60 random cells from each of three independent experiments were selected for analysis. FNDs were detected at λ_ex/em_ = 561/659 nm, Dio-oxLDL at λ_ex/em_ =495/510 nm, and Lysoview 405 at λ_ex/em_ =358/461 nm. The JAcoP plugin in FIJI was employed to determine whether FNDs colocalized with Lysoview 405 and whether Dio-oxLDL colocalized with Lysoview 405 (<https://imagej.nih.gov/ij/plugins/track/jacop.html>). The Manders’ Coefficient (MCC), a widely used metric for organelle colocalization analysis, indicated the overlapping fraction of different compartments^6^. Thresholds of each channel were determined from the control group .

**Cell viability test**

To assess the cell viability, the CellTiter-Glo Luminescent Cell Viability Assay from Promega was used, measuring ATP levels as an indicator of metabolically active cells. Macrophages were seeded in clear flat-bottom 96-well plates at a density of 50,000 cells/well. After removing the cell culture medium, cells were rinsed once with PBS.

For the FND biocompatibility test, cells were incubated with either 10 µg/mL FNDs or 5% Dimethyl sulfoxide (DMSO) as a positive control for 24 hours. For the oxLDL cytotoxicity test, cells were incubated with 50 µg/mL oxLDL for varying durations (0, 0.5, 1, 2, 4, 6, or 8 hours) at 37°C with 5% CO_2_.

After the incubation period, the plate and its contents were equilibrated to room temperature for approximately 30 minutes. Then, 100 µL of CellTiter-Glo Reagent was added to 100 µL of medium-containing cells, and the mixture was thoroughly mixed for 2 minutes on an orbital shaker. The plate was incubated at room temperature for 10 minutes to stabilize the luminescent signal. Luminescence was measured using a FLUOstar Omega Microplate Reader (BMG Labtech, De Meern, The Netherlands), with cells that had only the medium changed serving as the baseline.

**ROS detection by Dihydroethidium (DHE) assay and MitoSOX red assay**

RAW 264.7 macrophages were seeded in a 35mm Petri dish at a density of 50,000 cells/mL and incubated with 50 µg/mL oxLDL for 0, 0.5, 1, 2, 4, 6, or 8 hours at 37°C with 5% CO_2_. After incubation, cells were washed with PBS. A solution of DHE (2 µg/mL) in DMEM medium was then added to the cells to detect reactive oxygen species (ROS) like intracellular superoxide. Cells were incubated for an additional 10 minutes at 37°C with 5% CO_2_. Fluorescence, indicative of ROS generation, was measured using a Leica Sp8x confocal microscope at excitation and emission wavelengths of 514 nm and 550-600 nm, respectively. Cells that had only the medium changed serving as the baseline.

For MitoSOX red, macrophages were seeded in 35mm petri dish at a density of 50,000 cells/well. After cells were incubated with 50 µg/mL oxLDL for 0, 0.5, 4 and 8 hours at 37°C with 5% CO_2_, 1uM of MitoSOX red was added into medium, incubating 30 mins, then imaged by Leica Sp8x confocal microscope with absorption/emission ∼396/610 nm.

To analyze fluorescence intensity by FIJI, the circle ROI tool was used to outline the desired regions. Area, Integrated Density, and Mean Grey Value were measured. Background fluorescence was determined from the control cell group and corrected as the baseline for calculating the final mean optical intensity. Thresholds of each channel were determined from the control and used in the analysis.

**TFEB, LAMP1 and RAB11a expression and quantification**

50,000 cells/mL were seeded in 35 mm glass-bottom Petri dishes and incubated with 50 µg/mL oxLDL for various times (0, 0.5, 1, 2, 4, 6, or 8 hours) at 37°C with 5% CO_2_. Following incubation, cells were fixed with 3.7% formaldehyde for 10 minutes. To assess TFEB expression, cells were stained using a rabbit anti-TFEB monoclonal primary antibody (Abcam, UK) or rabbit anti-RAB11a monoclonal primary antibody (Abcam, UK). A goat anti-rabbit IgG-Alexa 594 secondary antibody (Abcam, UK) were used as reporter. Nuclei were stained with DAPI. Z-stack confocal images covering the entire cell volume were acquired using a Leica SP8x confocal microscope. Alexa 594 was detected at λ_ex/em_ = 561/630 nm, and DAPI was imaged at 358/461 nm. LAMP1 was stained by Rabbit-anti-LAMP1 (L1418, Sigma-Aldrich), labelled by sencondary antibody goat-anti-rabbit Alexa 594.

To analyze TFEB, LAMP1 or RAB11a fluorescence intensity in different cell regions using FIJI, the circle ROI tool was used to outline the desired regions. Area, Integrated Density, and Mean Grey Value were measured. Background fluorescence was determined from the control cell group and corrected as the baseline for calculating the final mean optical intensity. Colocalization of nuclei and TFEB was analyzed using the JAcoP plugin. Thresholds of each channel were determined from the control and used in the analysis.

**Western blot**

For Western blot analysis, sample aliquots were combined with 2× sample buffer. The samples were then loaded onto a 10% SDS-PAGE gel and electrophoresed at 110 V. Proteins were transferred onto a nitrocellulose membrane (PerkinElmer, USA), blocked with 10% non-fat milk, and incubated overnight at 4°C with primary antibodies: anti-GAPDH (Sigma), anti-TFEB (Abcam), or anti-RAB11a (Abcam).

The following day, membranes were incubated with HRP-conjugated secondary antibodies (GE Healthcare) and detected using enhanced chemiluminescence with a ChemiDoc Imaging System (Bio-Rad). Signal intensities were quantified using ImageJ, and TFEB and RAB11a expression levels were normalized to GAPDH. Data analysis was performed using GraphPad Prism, with results presented as the average of three independent experiments.

**Free radical measurements in macrophages by T1 relaxometry**

A custom-built magnetometry setup, previously described, was used for T1 measurements^7^. This setup integrates a confocal microscope with an acousto-optical modulator (Gooch & Housego, model 3350-199) for detection. T1 relaxometry utilizes NV centers in diamonds to sense magnetic noise at room temperature. These NV centers measure the surrounding magnetic noise, including contributions from free radicals.

In T1 relaxometry, NV centers are initially excited into the bright ms=0 state. Measurements are taken at various intervals to determine the time it takes the NV centers to return to an equilibrium between ms=0 and ms=±1. The pulse sequence involves a series of 5 µs green laser pulses (561 nm) with a dark time (τ) ranging from 200 ns to 10 ms between them. The resulting brightness data from the initial 0.6 µs of each pulse is plotted against the dark time to generate relaxometry curves (Figure 1b). T1 is calculated using a bi-exponential model with short *T_S_* and long *T_L_* components, where *T_L_* represents the final T1 value, as shown in Eq. (1):

$I\left( \tau\right)=I_{\infty}(1+C_{s}e^{-\tau/Ts}+C_{L}e^{-\tau/TL})$ **(1)**

where, the photoluminescence intensity at long dark times (τ), denoted as I_∞_, reflects the final thermal equilibrium, and *C_S_* and *C_L_* represent the short and long contrast components of the relaxation curves, respectively^8,9^. The time required to reach the equilibrium decreases in the presence of free radicals. Each measurement involved repeating the pulsing sequence 10,000 times to ensure a high signal-to-noise ratio. The laser power at the sample was set to 50 µW to balance minimal cell damage with effective NV center polarization.

To study free radical generation in macrophage lysosomes, Raw 264.7 macrophages were seeded at 50,000 cells/mL in 35 mm glass-bottom Petri dishes and incubated overnight. After cell attachment, 50 µg/mL oxLDL and 10 µg/mL FNDs were added. For single-FND tracking, T1 measurements were conducted immediately upon locating an FND particle in a cell, which was then monitored for 60 minutes. Non-oxLDL-treated cells served as controls. For multi-FND measurements, after incubating cells with 50 µg/mL oxLDL and 10 µg/mL FNDs for 0, 0.5, 1, 2, 4, 6, or 8 hours at 37°C with 5% CO_2_, T1 measurements were performed. A total of 15 FNDs from different cells per group were selected for analysis.

**Statistical analysis**

Data analysis was performed using GraphPad Prism version 8.0. Statistical significance was evaluated using Two-tailed Student’s unpaired t-test, one-way ANOVA (analysis of variance), or two-way ANOVA with Tukey’s multiple comparisons test, depending on the experimental design. The significance levels were determined by comparing experimental groups to the control group.

**Table S1.** Manders’ coefficient (± standard deviations) of FND with lysoview 405 or Dio-oxLDL with lysoview 405 after different incubation time (from Figure 5b).

| Time | FND/lysoview 405 | oxLDL/lysoview 405 |
| --- | --- | --- |
| 0.5h | 0.804±0.125 | 0.874±0.089 |
| 1h | 0.849±0.077 | 0.931±0.061 |
| 2h | 0.819±0.112 | 0.864±0.098 |
| 4h | 0.815±0.061 | 0.885±0.066 |
| 6h | 0.799±0.045 | 0.843±0.038 |
| 8h | 0.866±0.082 | 0.839±0.084 |


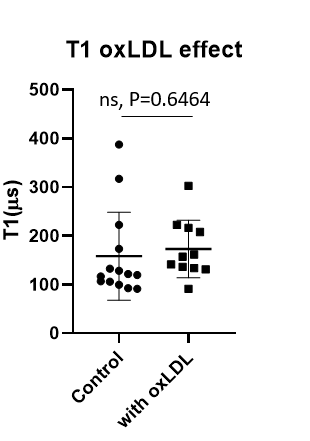


Figure S1, To study if oxLDL has effect on T1 measurement, 50 µg/mL oxLDL was added in FND-coated Petri dish, then T1 relaxometry was perofromed. Data was obtained from 3 independent experiments, analysed by T-test, and represented as mean ± SD.

Figure S2, Fluorescence graphs of individual channels of Figure 3d.


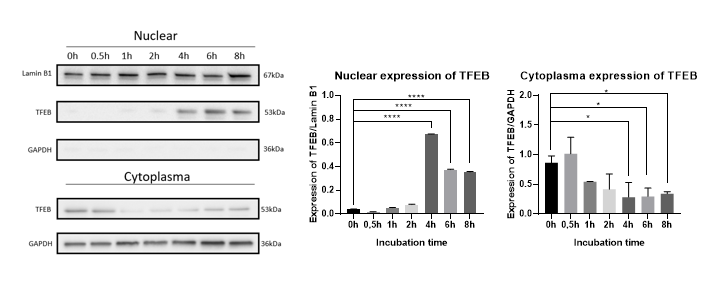


Figure S3, Western blot analysis of nuclear and cytoplasmic TFEB protein levels.


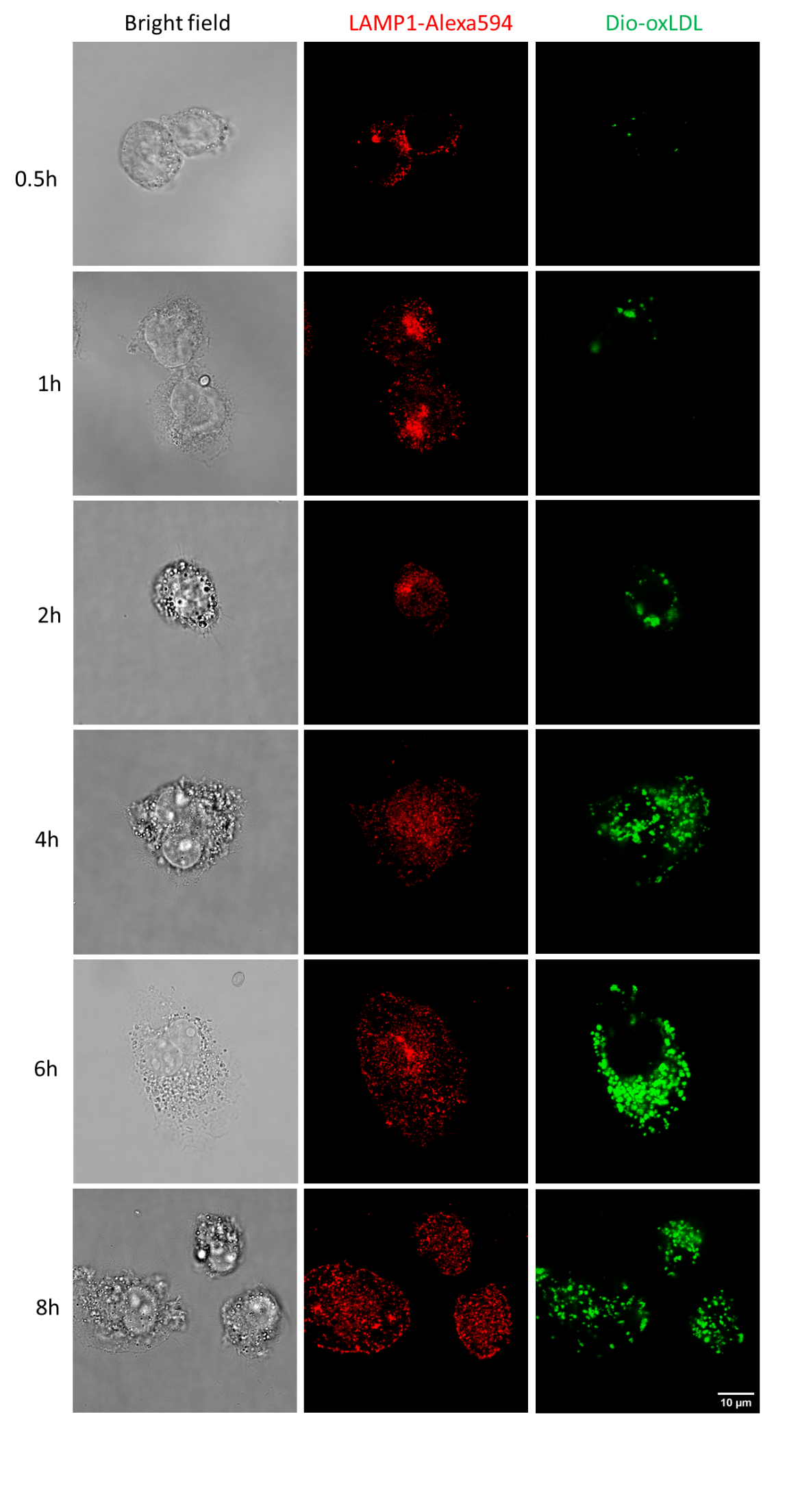


Figure S4, Fluorescence graphs of individual channels of Figure 3h.


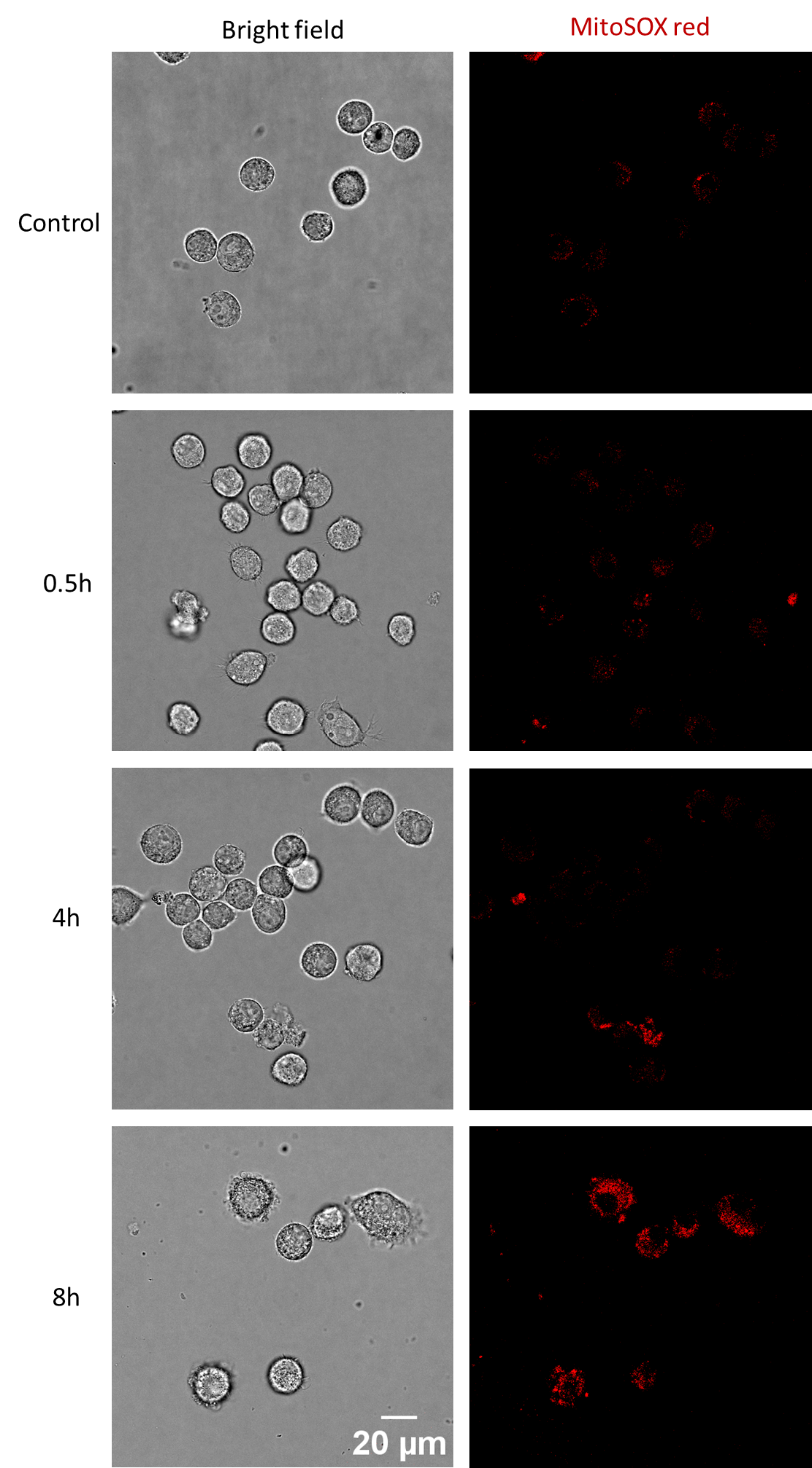


Figure S5, Fluorescence graphs of individual channels of Figure 4f.


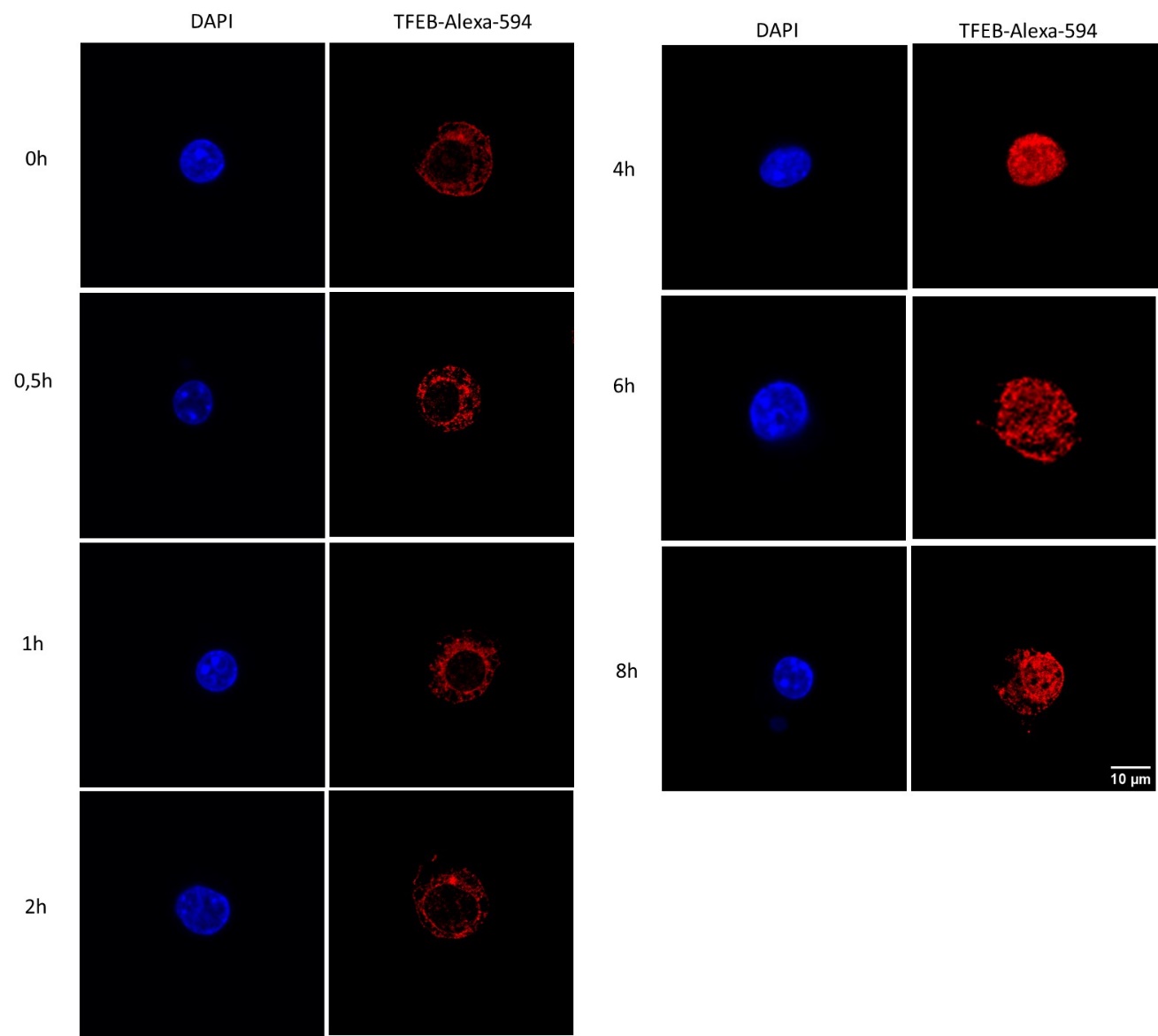


Figure S6, Fluorescence graphs of individual channels of Figure 5a.


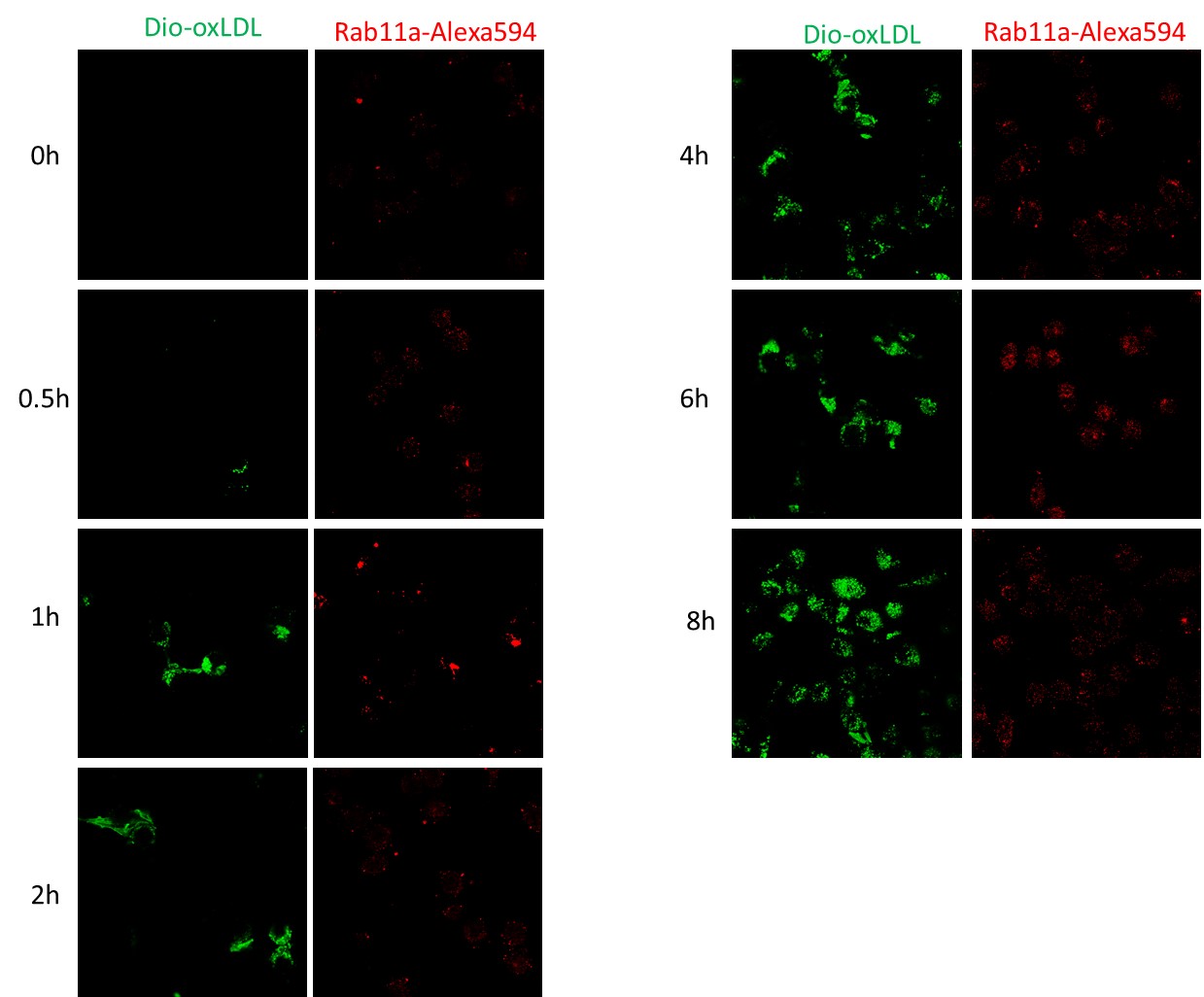


Figure S7, Fluorescence graphs of individual channels of Figure 5g.

Figure S8, T1 measurement that performed by FND-VDAC2, revealing the early mitochondrial ROS level.

Reference

(1) Shenderova, O. A.; Shames, A. I.; Nunn, N. A.; Torelli, M. D.; Vlasov, I.; Zaitsev, A. Review Article: Synthesis, Properties, and Applications of Fluorescent Diamond Particles. *Journal of Vacuum Science and Technology. B, Nanotechnology & Microelectronics* **2019**, *37* (3), 030802. https://doi.org/10.1116/1.5089898.

(2) Schirhagl, R.; Chang, K.; Loretz, M.; Degen, C. L. Nitrogen-Vacancy Centers in Diamond: Nanoscale Sensors for Physics and Biology. **2014**. https://doi.org/10.1146/annurev-physchem-040513-103659.

(3) Mochalin, V. N.; Shenderova, O.; Ho, D.; Gogotsi, Y. The Properties and Applications of Nanodiamonds. **2012**. https://doi.org/10.1038/NNANO.2011.209.

(4) Mohan, N.; Chen, C.-S.; Hsieh, H.-H.; Wu, Y.-C.; Chang, H.-C. In Vivo Imaging and Toxicity Assessments of Fluorescent Nanodiamonds in Caenorhabditis Elegans. **2010**. https://doi.org/10.1021/nl1021909.

(5) Nie, L.; Nusantara, A. C.; Damle, V. G.; Sharmin, R.; Evans, E. P. P.; Hemelaar, S. R.; van der Laan, K. J.; Li, R.; Perona Martinez, F. P.; Vedelaar, T.; Chipaux, M.; Schirhagl, R. Quantum Monitoring of Cellular Metabolic Activities in Single Mitochondria. *Sci Adv* **2021**, *7* (21), 573. https://doi.org/10.1126/SCIADV.ABF0573/SUPPL_FILE/ABF0573_SM.PDF.

(6) Dunn, K. W.; Kamocka, M. M.; McDonald, J. H. A Practical Guide to Evaluating Colocalization in Biological Microscopy. *Am J Physiol Cell Physiol* **2011**, *300* (4), C723. https://doi.org/10.1152/AJPCELL.00462.2010.

(7) Loretz, M.; Pezzagna, S.; Meijer, J.; Degen, C. L. Nanoscale Nuclear Magnetic Resonance with a 1.9-Nm-Deep Nitrogen-Vacancy Sensor. **2014**.

(8) Perona Martínez, F.; Nusantara, A. C.; Chipaux, M.; Padamati, S. K.; Schirhagl, R. Nanodiamond Relaxometry-Based Detection of Free-Radical Species When Produced in Chemical Reactions in Biologically Relevant Conditions. **2023**, *30*, 38. https://doi.org/10.1021/acssensors.0c01037.

(9) Vedelaar, T. A.; Hamoh, T. H.; Martinez, F. P. P.; Chipaux, M.; Schirhagl, R. Optimizing Data Processing for Nanodiamond Based Relaxometry. *Adv Quantum Technol* **2023**. https://doi.org/10.1002/QUTE.202300109.
